# Pulmonary endarterectomy for chronic thromboembolic pulmonary hypertension with active Crohn’s disease

**DOI:** 10.1186/s40792-019-0616-7

**Published:** 2019-04-11

**Authors:** Kayo Sugiyama, Shun Suzuki, Keita Maruno, Toshiki Fujiyoshi, Nobusato Koizumi, Hitoshi Ogino

**Affiliations:** 1 Department of Cardiac Surgery, Aichi Medical University Hospital, 1-1 Yazakokarimata, Nagakute, Aichi 480-1195 Japan; 2 Department of Cardiovascular Surgery, Tokyo Medical University Hospital, 6-7-1, Nishishinjuku, Shinjuju, Tokyo, 160-0023 Japan

**Keywords:** Chronic thromboembolic pulmonary hypertension, Pulmonary endarterectomy, Crohn’s disease, Inflammatory bowel disease, Balloon pulmonary angioplasty

## Abstract

**Background:**

Anticoagulation control in active inflammatory bowel disease (IBD) is challenging because of hypercoagulation and bleeding complications. The strategy for treating chronic thromboembolic pulmonary hypertension (CTEPH) in IBD remains controversial because only a few studies have reported its successful treatment (Kim and Lang. Eur Respir Rev 21: 27-31, 2012, Bonderman, et al. Circulation 115: 2153-8, 2007). We describe a case of CTEPH with active Crohn’s disease successfully treated with pulmonary endarterectomy (PEA).

**Case presentation:**

A 49-year-old man with CTEPH had undergone balloon pulmonary angioplasty four times; however, severe pulmonary hypertension remained. Moreover, he had Crohn’s disease, and sufficient anticoagulant therapy could not be performed because of frequent melena. He also had frequent episodes of intestinal ileus resulting in malnutrition. After strict anticoagulant control with warfarin, PEA was performed safely with strict control of the activated coagulation time. After PEA, his pulmonary hypertension improved to a normal range, and he underwent abdominal surgery for the recurrent intestinal ileus.

**Conclusion:**

PEA for CTEPH with active IBD is challenging, but feasible. The strict anticoagulant control is critical for active IBD patients. Safety of taking direct oral anticoagulants is unclear because there are no parameters for monitoring the level of anticoagulation.

## Background

The incidence of systemic thromboembolic events in inflammatory bowel disease (IBD) is higher than that in the general population, and IBD is now recognized as one of the risk factors for chronic thromboembolic pulmonary hypertension (CTEPH) [1, 2]. Anticoagulant therapeutic management in active IBD is controversial because of the complication of severe intestinal bleeding [3]. Furthermore, patients with active IBD should be considered compromised hosts with malnutrition. In accordance with these factors, pulmonary endarterectomy (PEA) for CTEPH in IBD poses a great challenge to surgeons [1, 4]. Balloon pulmonary angioplasty (BPA) has been administered more frequently preoperatively and postoperatively in patients with CTEPH; however, its effectiveness is limited to distal lesions. Here, we describe a case of CTEPH with active Crohn’s disease successfully treated by PEA.

## Case presentation

A 49-year-old man had been receiving medical treatment for CTEPH for 3 years. Although he had undergone BPA four times previously at another hospital before admission to our hospital, pulmonary hypertension persisted. He had also been treated for nontuberculous mycobacterial infection with antibiotics and Crohn’s disease with mesalazine (3000 mg/day). Despite this thorough treatment and strict nutritional control, he often experienced high fever and melena.

When he was admitted to our institution, his anticoagulant control was not sufficient because of malnutrition and bleeding complications. Since admission, he repeatedly developed high fever and melena owing to active Crohn’s disease. Given that melena occurred more frequently after the administration of direct oral anticoagulant (DOAC), the DOAC treatment was switched to anticoagulant therapy using warfarin with strict control of the low-level prothrombin international normalized ratio (range 1.5–2.0). Subsequently, melena was relieved. Two weeks before PEA, because of frequent intestinal ileus, he was placed on liquid nutritional supplementation to avoid intestinal inflammation.

**Fig. 1:**
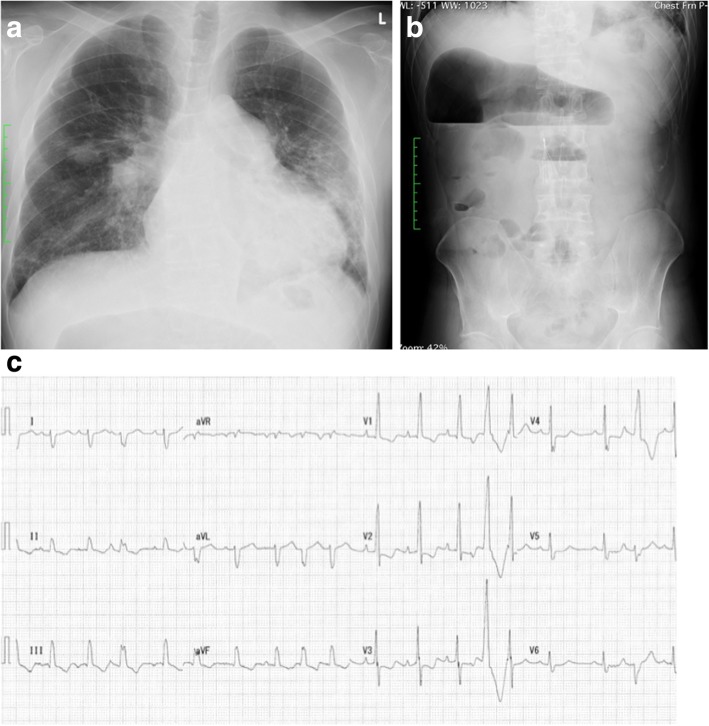
**a** Preoperative chest radiograph showing cardiomegaly involving the right cavities and notable enlargement of the pulmonary arch. **b** Preoperative abdominal radiograph showing chronic intestinal paralysis. **c** Preoperative electrocardiogram showing a right bundle branch block and right ventricular hypertrophy

**Fig. 2:**
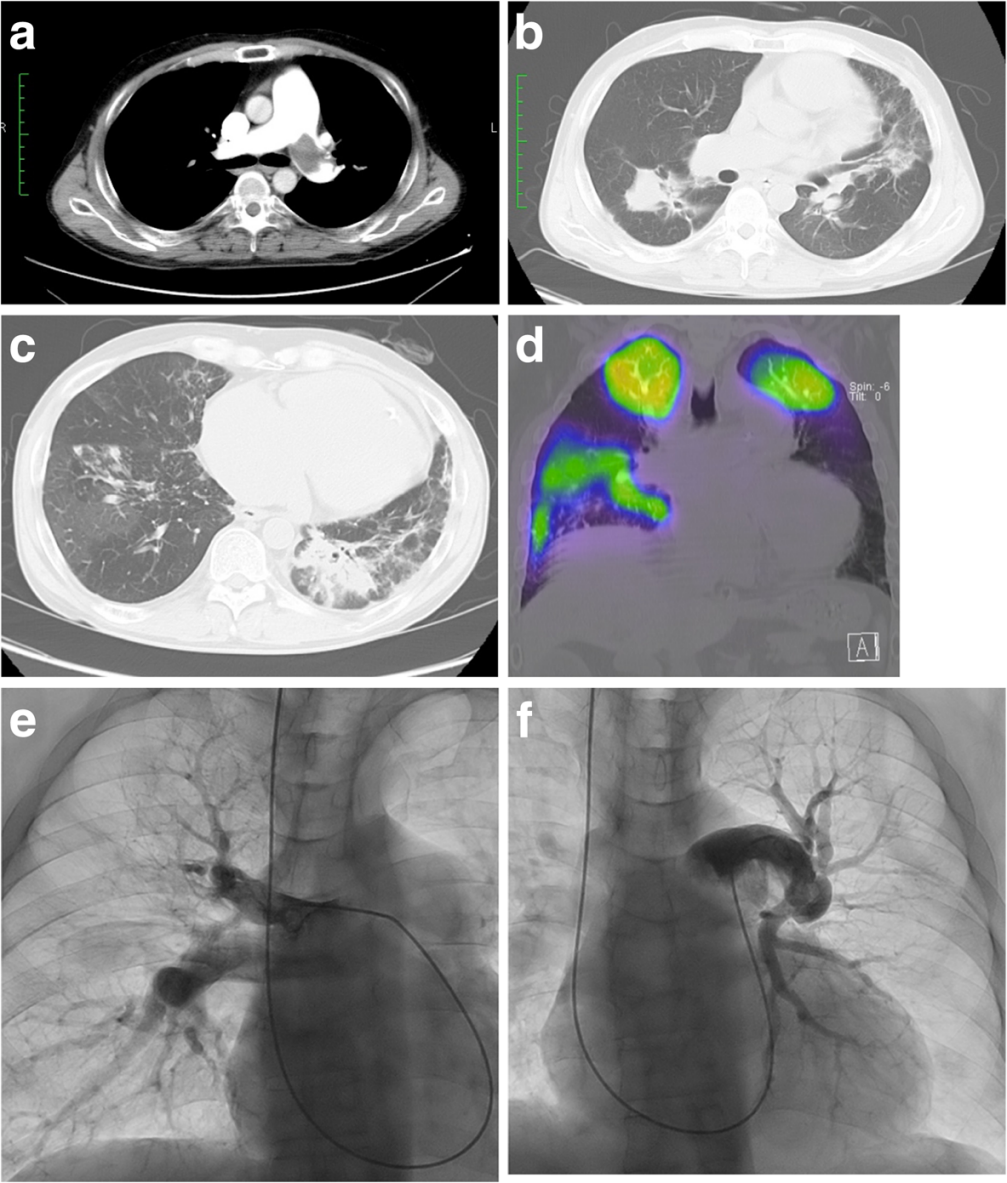
**a** Preoperative computed tomography scan of the lung showing showed proximal chronic pulmonary emboli. **b** Preoperative computed tomography scan of the lung showing a nontuberculous mycobacterial infection. **c** Preoperative computed tomography scan of the lung showing aspiration pneumonia. **d** Preoperative lung perfusion scintigraphy showing multiple segmental defects. **e**, **f** Preoperative pulmonary artery angiograms showing intimal irregularities and abrupt narrowing of both pulmonary arteries

The preoperative laboratory data indicated mild inflammatory changes, moderate chronic kidney disease, low-level protein, and anemia, with the following results: white blood cell count, 3.1 × 10^3^/μL; C-reactive protein level, 2.0 mg/dL; creatinine level, 1.8 mg/dL; estimated glomerular filtration rate, 33 mL/min/1.73 m^2^; albumin level, 2.8 g/dL; and hemoglobin level, 7.1 g/dL. He had no risk factors of thrombophilia. Chest radiograph showed cardiomegaly involving the right cavities and notable enlargement of the pulmonary arch (Fig. 1a). Abdominal radiograph showed a large amount of gas in the colon due to chronic intestinal paralysis (Fig. 1b). Electrocardiogram showed a right bundle branch block and signs of right ventricular hypertrophy (Fig. 1c). Echocardiogram showed severe left ventricular compression due to dilated right ventricle, and the estimated right ventricular pressure was 105 mmHg. Venous echosonogram showed old thrombi in both femoral veins. Computed tomography scan of the lung showed proximal chronic pulmonary emboli (Fig. 2a), infiltrations because of nontuberculous mycobacterial infection (Fig. 2b), and recent aspiration pneumonia (Fig. 2c). Lung perfusion scintigraphy revealed multiple segmental defects (Fig. 2d), and pulmonary arterial angiography showed a thickened proximal pulmonary arterial intima, intimal irregularities, and abrupt narrowing of both pulmonary arteries (Fig. 2e, f). Findings of the right heart catheter examination indicated severe pulmonary hypertension with the following parametric values: pulmonary artery pressure, 96/32 mmHg (mean, 53 mmHg); calculated total pulmonary resistance, 1247 dynes/sec/cm^−5^; and pulmonary vascular resistance, 1012 dynes/sec/cm^−5^.

**Fig. 3:**
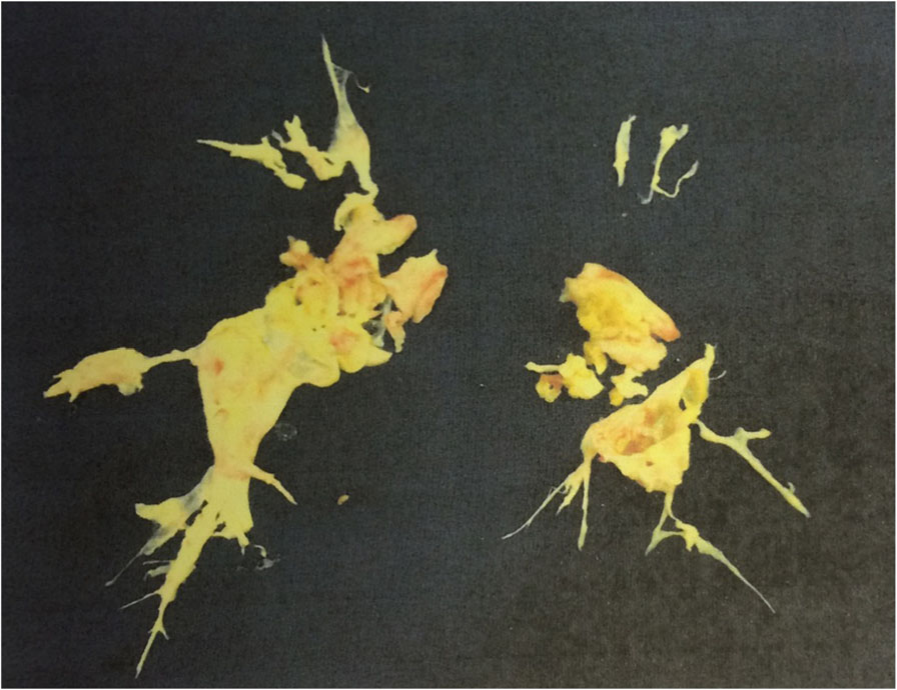
Resected thromboembolism of both pulmonary arteries

After undergoing thorough treatments for active IBD, PEA using intermittent circulatory arrest under deep hypothermia was performed (Fig. 3). PEA was performed through a median sternotomy using cardiopulmonary bypass with deep hypothermic intermittent circulatory arrest, similar to the techniques established by Jamieson et al. [5]. During PEA, the activated clotting time was strictly controlled between 350 and 500 s. Weaning from cardiopulmonary bypass was uneventful, and pulmonary hypertension improved dramatically to the following parametric values: mean pulmonary artery pressure, 16 mmHg; calculated total pulmonary resistance, 659 dynes/sec/cm^−5^; and pulmonary vascular resistance, 589 dynes/sec/cm^−5^. After PEA, anticoagulant therapy using warfarin was resumed with low-level control of the prothrombin international normalized ratio (range 1.5–2.5). Unfortunately, because mild melena and ileus occurred 2 weeks after PEA, he was placed on liquid nutritional supplementation again. Furthermore, the treatment for the mild mediastinitis with lavage and closure of the wound was needed.

Subsequently, the patient recovered completely and was transferred to the previous hospital to undergo surgical treatment for recurrent ileus. After subtotal colectomy, he was still doing well without any cardiovascular events during the 2-year follow-up.

## Discussion

Thromboembolic complications in IBD were first described in 1936 [3]. Although deep vein thrombosis of the lower limbs and pulmonary embolism are the most common thromboembolic phenomena, thromboembolic complications in cerebral and retinal vessels, peripheral arteries, and portal and mesenteric veins have been reported [6, 7]. CTEPH has also been reported as a complication of IBD [1, 2]. Some factors including IBD have been recognized as risk factors for CTEPH development [1, 2, 4].

Active inflammation is associated with the onset of coagulability. Cytokines play a primary role in the pathogenesis of IBD and have a significant effect on the coagulation cascade. The interaction between inflammation and hemostasis affects the coagulation mechanism and platelet function [8–10]. The present patient had suffered from multiple diseases including active IBD, nontuberculous infection, and aspiration pneumonia resulting in active inflammation.

Spontaneous bleeding complications in active IBD make it difficult to treat thromboembolic phenomena. Although unfractionated heparin has been suggested for treating venous thrombosis in IBD, its role is controversial. Massive hemorrhage which requires emergency colectomy is seen in 3% of these patients [3]. The safety of taking DOACs is also unclear because there are no parameters for monitoring the level of anticoagulation and there are no antagonists to reverse its effects. In the present case, frequent melena occurred after the administration of DOAC and the symptom was relieved after changing the anticoagulant therapy to warfarin with low-level prothrombin international normalized ratio control. Furthermore, strict control of the activated clotting time in a limited level during PEA prevented critical bleeding complications. Even after PEA, strict anticoagulation control using warfarin was necessary.

The patient in the present case frequently experienced melena and ileus. Thus, adequate nutritional replenishment including total parenteral nutrition should be considered in this case during the perioperative period. Despite these treatments, the patient showed malnutrition which made the anticoagulant therapy difficult. Infection control is also essential, because malnutrition is related to compromised host, and bacterial translocation occurs easily due to repeated intestinal paralysis in active IBD patients.

Additionally, BPA has been known as an effective endovascular treatment for CTEPH preoperatively and postoperatively; however, its effectiveness is limited to only distal lesions, and PEA is the most appropriate treatment for CTEPH with central lesions [11]. In the present case, BPA performed four times was not effective, and only PEA improved his severe pulmonary hypertension to almost normal levels.

## Conclusions

Performing PEA for CTEPH with active IBD is still challenging but feasible. Although the safety of taking DOACs is still unclear, strict anticoagulation control and multidisciplinary treatment including nutrition and infection control are also critical.

## Abbreviations

BPA: Balloon pulmonary angioplasty
CTEPH: Chronic thromboembolic pulmonary hypertension
DOAC: Direct oral anticoagulant
IBD: Inflammatory bowel disease
PEA: Pulmonary endarterectomy acquisition
